# Dental Periostitis

**Published:** 1872-10

**Authors:** J. A. Stipp


### Dental Periostitis.

*Read Before the Mad River Dental Society.*

BY J. A. STIPP, D. D. S.

*Mr. President, and Gentlemen:* Having been appointed as one among others to prepare a paper for this meeting, allow me for a very brief season to occupy your valuable time in the perusal of a few thoughts suggested to me upon the subject of Dental Periostitis. I do not for one moment think myself capable or competent to portray or say anything whatever, that will have a tendency to enlighten you upon this subject—so much having been said and written, that what I may here say will be of little or no consequence. But as it is one of the subjects presented in our programme, and to be discussed here at this time, I hope that it may elicit something, and in a manner vie with the thoughts of you, my older and by far more *experienced* friends. In the first place let us consider the structure of the periosteum; second, the signs or indications of periostitis; third, its cause, and finally its treatment. Investing roots of the teeth, in fact all bony structures, we find a white dense fibrous membrane, the periosteum, to which it is intimately attached by fibrous prolongations, and numerous minute blood vessels.

It is highly vascular, and consists of two layers closely united together, one formed chiefly of connective tissue, the other of elastic fibres of a finer kind which form a dense, elastic membranous net-work.

It completely invests the roots of the teeth as far as their necks, and at the margin of the alveolus becomes continuous with the fibrous structure of the gums.

It has been maintained by some that it is throught his medium that the tooth receives its nourishment after the death and loss of the *pulp*.

The signs, or at least the first indications we have of this disease, is a slight fulness, which invites contact and even pressure from the opposing teeth, such pressure affording rather a pleasurable sensation and seeming relief. This condition is induced by such irritating causes as would produce inflammation in any of the other soft tissues, and is the result of a determination of blood to the part. The surrounding parts being very strong prevents the expansion of the capillaries and other small vessels ramifying this membrane. The walls being pressed upon in proportion to the force or extent of this determination, this effort of expansion will occasion, especially in those teeth having conical roots, a slight elongation, and more particularly when active inflammation supervenes. After active inflammation has occurred, pressure or tapping upon the affected tooth will not unfrequently cause great pain. This condition varies in degree in different cases, depending largely upon the predisposition and susceptibility to exciting causes of irritation and inflammation, together with the character of these exciting causes, whether concentrated in action to a mere point or more extended in their sphere of operation.

Many times only a small portion of the periosteum of a tooth will be thus affected; tapping on one side of the affected tooth will occasion pain, while if the other side is subjected to the same treatment scarcely any pain or sensation of uneasiness is at all perceptible.

this In affection it is often found that the periosteum will, on one side of the tooth pass through the successive stages of inflammation to destruction, while on the other side of the tooth it has suffered nothing more than a slight irritation: In such cases the part is endowed with such a degree of vitality that it is able to overcome this attack and thereby confine it to a certain locality. When there is a predisposition, the local exciting causes will sooner and more vigorously make the attack; thus, when the pulp of a tooth is devitalized, or its life so much impaired that it is unable to perform its function, the periosteum is far more liable to disease, for several reasons.

In many cases there are irritants at hand that did not exist before, and the periosteum is either enfeebled and not able to offer resistance, or the demand upon its function greater than before, it being the medium of connection between the *normal vital* tissue and that which is devitalized.

And when this condition exists it is wholly through the periosteum that the tooth is nourished, if at all. Now if we accept this as *true*, we may then conclude that there is a greater susceptibility on the part of the periosteum after the death of the pulp than before.

One of the exciting causes of this affection is found in the acrid debris of the dead and decaying pulp of the tooth, passing out through its foramen, either in a fluid or gaseous state, there coming in contact with the periosteum. Another is, deposits, calcareous and others, are insinuated beneath the margin of the gum, there encroaching upon and irritating the periosteum to such an extant as to produce this state of affairs.

It is sometimes occasioned by an extension of inflammation from another part; the periosteum of one tooth may be affected to such an extent that one or more of the neighboring teeth may be affected by the extension of such inflammation.

Some medicinal agents produce a similar condition by thickening the tissues and elongating the teeth. Mercurials, for instance, present a fine illustration of this class. Not yielding in the slighest degree to those remedies that in many other cases accomplish all that could be desired, the treatment of periostitis in its details, whether local or systemic, will be governed by the attendant conditions, such as systemic predispositions, the vital force, the local causes and their peculiarities.

Systemic treatment should have for its object the removal or counteracting of predispositions and the abatement of the determination of blood to the part by inviting it to other parts, such as will have a tendency to allay the excitement in the part affected, and as far as possible restore the equilibrium of the circulation. The local remedies also must be judiciously and faithfully applied. The same principles applicable in the treatment of inflammation in any of the other soft tissues are the same to be employed here. Many for the want of adaptability have to be thrown aside. But we have, however, at our command some very active and efficient remedies for the treatment of this affection.

The causes producing and influencing this disease should always be fully apprehended, after which the following points should receive due consideration: First, remove the dead pulp and all of the dead and broken-down tissue; cleanse the canal or canals in the root of the tooth; remove the tartar from the necks of the teeth, rendering them perfectly free from all local exciting causes. Extract all dead roots in the vicinity of the affected tooth. Second, by relieving the congestion in the affected part, either by systemic treatment or by counter-irritation.

Counter-irritation may be produced by depletion, scarify- the gum, or by the application of some counter-irritating agent, such as the tincture of iodine or capsicum, or cantharidal collodion. The action ot any of these agents will produc- the desired effect. The tincture of iodine, a very valuable and favorite remedy with many, is easily applied, and will produce the most glorious result. In the treatment of periostitis in dead teeth, cleanse the canal well as possible; moisten a plegget of cotton with the tincture of iodine and carbolic acid, pass it into the canal, protecting it with sandarac varnish. Apply tincture of iodine to the gum of the affected tooth, repeating this treatment from day to day until your are satisfied that it is restored to its normal condition. Then moisten a very small plegget of cotton with carbolic acid, pass it up the canal to the apex of the root, having in the meantime prepared the cavity; introduce the filling, making it as *compact* as possible, and success will crown your every effort in almost every instance.

Gentleman, I hope you will accept these few thoughts, simply as *suggestions, and not as stubborn facts.*

